# Supplementary material for: Transcriptional and epigenetic rewiring by the NUP98::KDM5A fusion oncoprotein directly activates CDK12
Source: Nat Commun. 2025 May 19;16:4656. doi: 10.1038/s41467-025-59930-9 (PMC12089343; doi:10.1038/s41467-025-59930-9)

Inventory of Supplementary Information

- Supplementary Figures and Supplementary Figure Legends
- Uncropped scans of Supplementary Figures 2H and 3B

Supplementary Figure 1

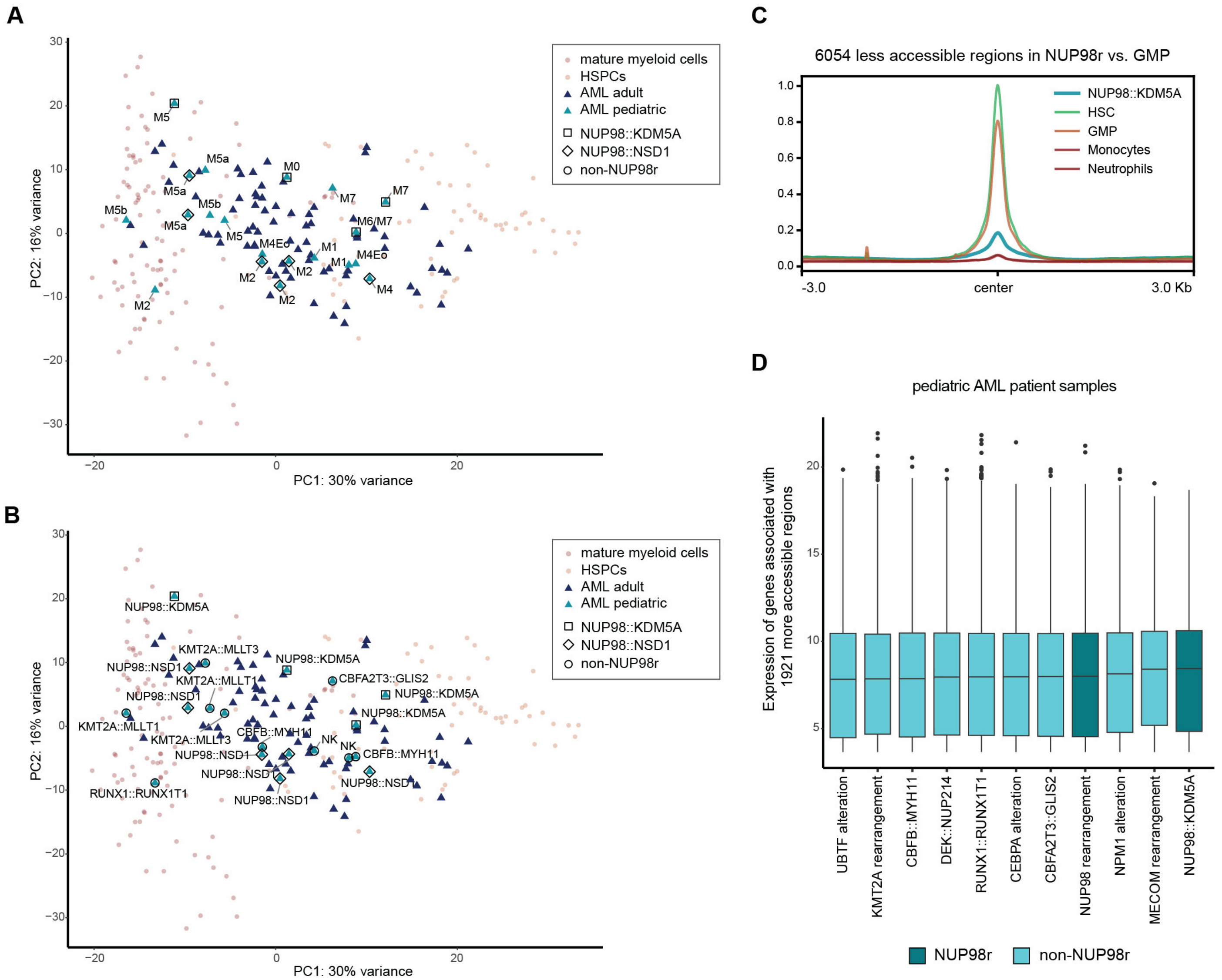

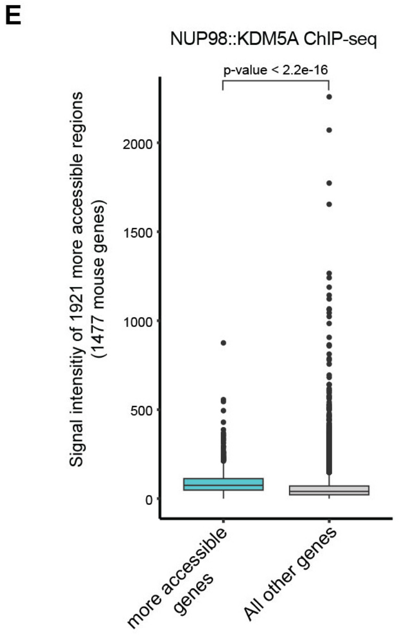

F

top *de novo* motifs in 1921 more accessible regions:

Rank	Motif	p-value	% of targets	% of background	best match (TF family)
1	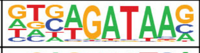	1e-152	30.35%	9.08%	GATA-2 (GATA)
2	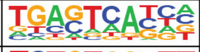	1e-48	10.36%	3.06%	JunB (AP-1)
3	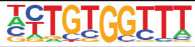	1e-39	33.63%	20.66%	RUNX1 (RUNX)
4	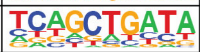	1e-23	10.67%	4.95%	TFAP4 (bHLH-ZIP)

top *de novo* motifs in 6054 less accessible regions:

Rank	Motif	p-value	% of targets	% of background	best match (TF family)
1	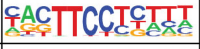	1e-747	35.86%	8.64%	PU.1 (ETS)
2	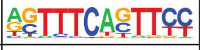	1e-307	9.53%	1.23%	IRF8 (IRF)
3	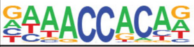	1e-251	24.25%	9.36%	RUNX1 (RUNX)
4	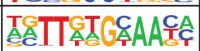	1e-97	6.51%	1.84%	CEBPD (C/EBP)

Supplementary Figure 1. Epigenomic analysis of AML patient data reveals *NUP98* fusion-specific patterns

(A) Principal component analysis of ATAC-seq data with FAB type and **(B)** fusion oncoprotein annotation for pediatric AML samples. **(C)** ATAC-seq profile plots showing 6054 less accessible regions in *NUP98* fusion-expressing AML vs. GMPs. **(D)** Expression of genes associated with the 1921 more accessible regions in pediatric AML patients from the St. Jude Cloud data repository. Data are presented as boxplots where the center line represents the median, the bounds of the box indicate the first (25th percentile) and third (75th percentile) quartiles and the whiskers extend to 1.5 * inter-quartile range from the hinges. Data points beyond this range are shown as individual dots and represent outliers. **(E)** ChIP-seq signal of NUP98::KDM5A chromatin binding at mouse homologs of genes associated with the 1921 more accessible regions (Wilcoxon rank sum test with continuity correction). Data are presented as boxplots where the center line represents the median, the bounds of the box indicate the first (25th percentile) and third (75th percentile) quartiles and the whiskers extend to 1.5 * inter-quartile range from the hinges. Data points beyond this range are shown as individual dots and represent outliers. **(F)** *De novo* motif enrichment analysis of transcription factor binding motifs in the 1921 more and 6054 less accessible regions in *NUP98*-rearranged AML vs. GMP. (HSPC, Hematopoietic Stem and Progenitor Cell, HSC, Hematopoietic Stem Cell, GMP, Granulocyte-Monocyte Progenitor). Source data are provided as a Source Data file.

Supplementary Figure 2

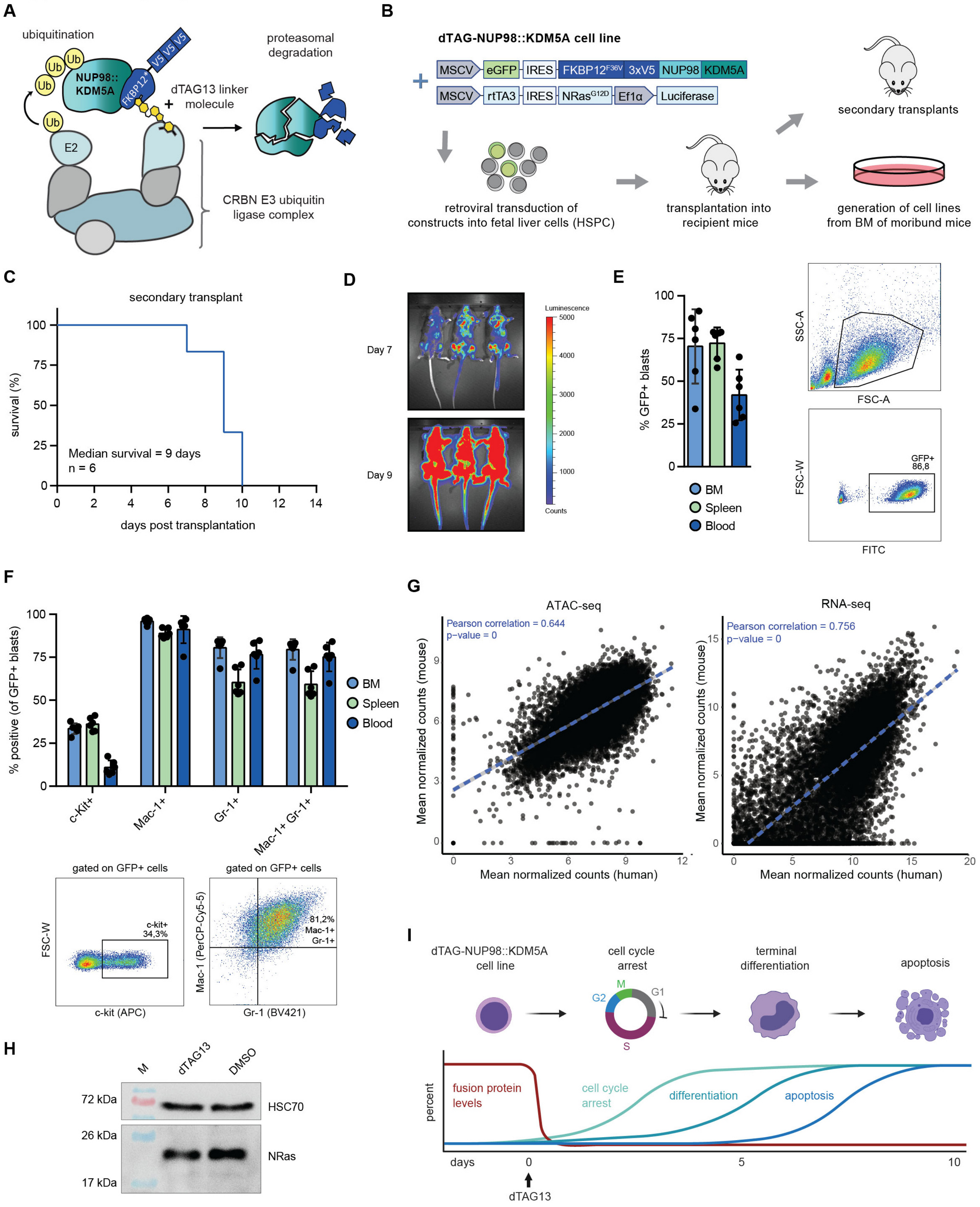

Supplementary Figure 2. Establishment of a ligand-inducible NUP98::KDM5A degradation AML model

(A) Schematic overview of the dTAG-NUP98::KDM5A model. **(B)** Scheme of used constructs and development of the dTAG-NUP98::KDM5A model. **(C)** Kaplan-Meier curve of recipient mice transplanted with dTAG-NUP98::KDM5A + *Nras*^{G12D} fetal liver cells (n = 6). **(D)** Live-imaging of bioluminescence signal in recipient mice transplanted with dTAG-NUP98::KDM5A + *Nras*^{G12D} fetal liver cells at day 7 and day 9 post transplantation. **(E)** Flow cytometric analysis of bone marrow, spleen and blood from moribund mice showing the percentage of GFP-positive blasts (n = 6, mean ± SD) **(F)** and the percentage of c-Kit, Mac-1 and Gr-1 surface marker-expressing GFP-positive blasts including representative flow cytometry plots (n = 6, mean ± SD). **(G)** Pearson correlation of ATAC-seq and RNA-seq data between NUP98::KDM5A patient samples and dTAG-NUP98::KDM5A cells. **(H)** Western blot analysis of dTAG-NUP98::KDM5A cells treated with dTAG13 (35 nM, 24h) using antibodies against NRas and HSC70. M = protein marker, (n = 1). **(I)** Schematic overview summarizing the data from cell cycle, differentiation and apoptosis analysis over time after dTAG13-induced degradation of NUP98::KDM5A. (CRBN, cereblon, HSPC, Hematopoietic Stem and Progenitor Cell, BM, bone marrow, kDa, kilodalton, SSC-A, side scatter area, FSC-w, forward scatter width, FITC, Fluorescein isothiocyanate). Parts of the figure were created in BioRender. Grebien, F. (2025) *https://BioRender.com/n74j643.* Source data are provided as a Source Data file.

Supplementary Figure 3

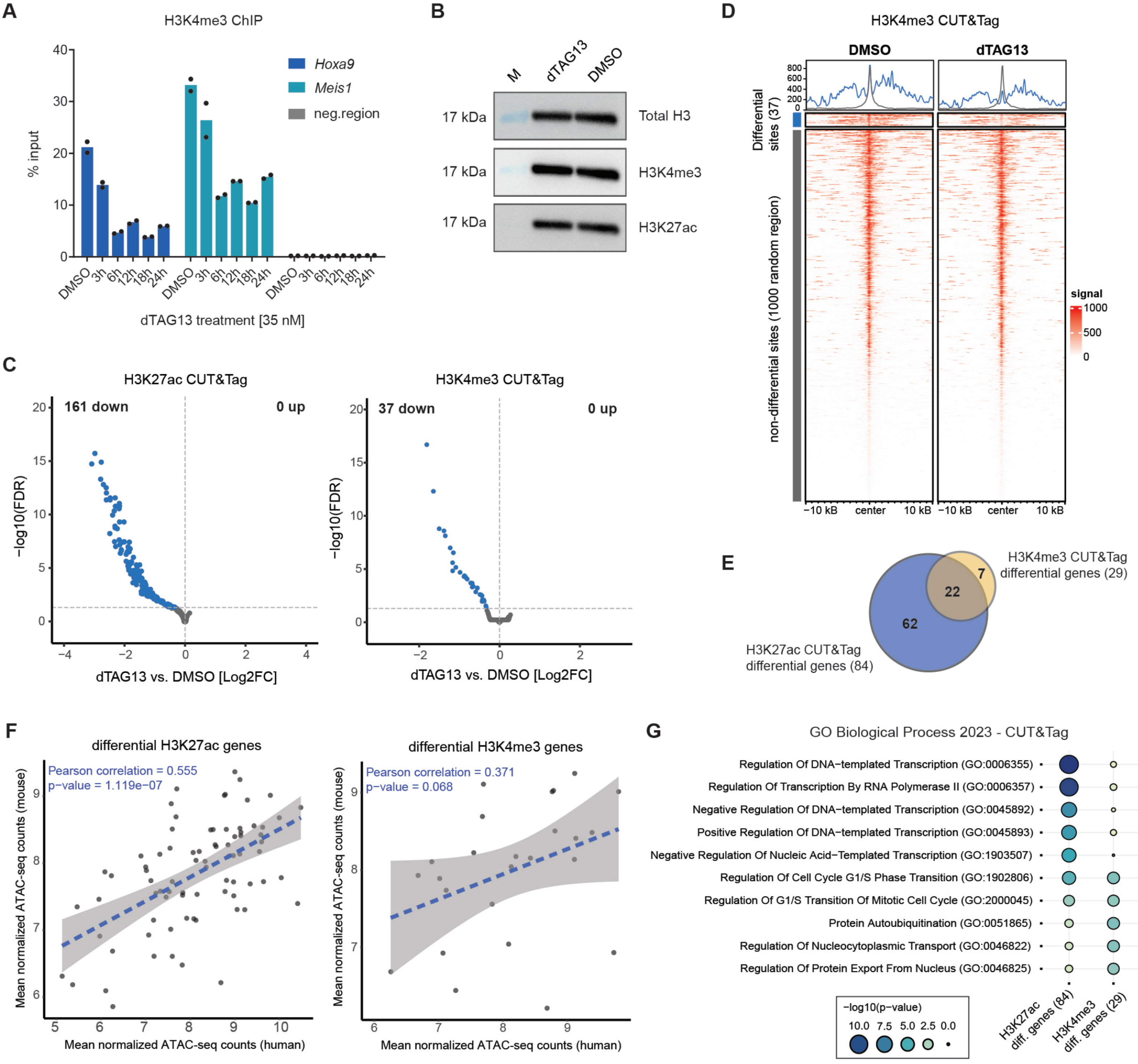

Supplementary Figure 3. NUP98::KDM5A maintains activating H3K27ac and H3K4me3 marks

(A) H3K4me3 ChIP-qPCR in dTAG-NUP98::KDM5A cells for *Hoxa9* and *Meis1* promoter regions and a negative region after treatment with dTAG13 for indicated time points compared to DMSO (n = 2 technical replicates). **(B)** Western blot analysis of dTAG-NUP98::KDM5A cells treated with dTAG13 (35 nM, 24h) using antibodies against total H3, H3K27ac and H3K4me3. M = protein marker, (n = 1). **(C)** Volcano plot of H3K27ac and H3K4me3 CUT&Tag data in dTAG-NUP98::KDM5A cells showing log2FC and –log10(FDR) values after 8h of dTAG13 treatment compared to DMSO (n = 3, statistical analysis and p-value calculations were performed using DiffBind). (**D)** Heatmaps and profile-plots of H3K4me3 CUT&Tag data of dTAG13 (35 nM) and DMSO-treated dTAG-NUP98::KDM5A cells showing significantly downregulated sites compared to 1000 random sites with no significant changes (n = 3, FDR < 0.05). **(E)** Intersection of CUT&Tag data showing the overlap of genes with differential H3K27ac and H3K4me3 signals after dTAG13 treatment compared to DMSO. **(F)** Pearson correlation of ATAC-seq data between NUP98::KDM5A patient samples and dTAG-NUP98::KDM5A cells showing the 84 differential H3K27ac and 29 differential H3K4me3 genes (the dashed line represents the linear regression fit and the shaded region around the line shows the 95% confidence interval). **(G)** Gene ontology analysis of CUT&Tag data showing top enriched terms of genes with differential H3K27ac and H3K4me3 signals after dTAG13 treatment compared to DMSO (GO Biological Process 2023). Source data are provided as a Source Data file.

Supplementary Figure 4

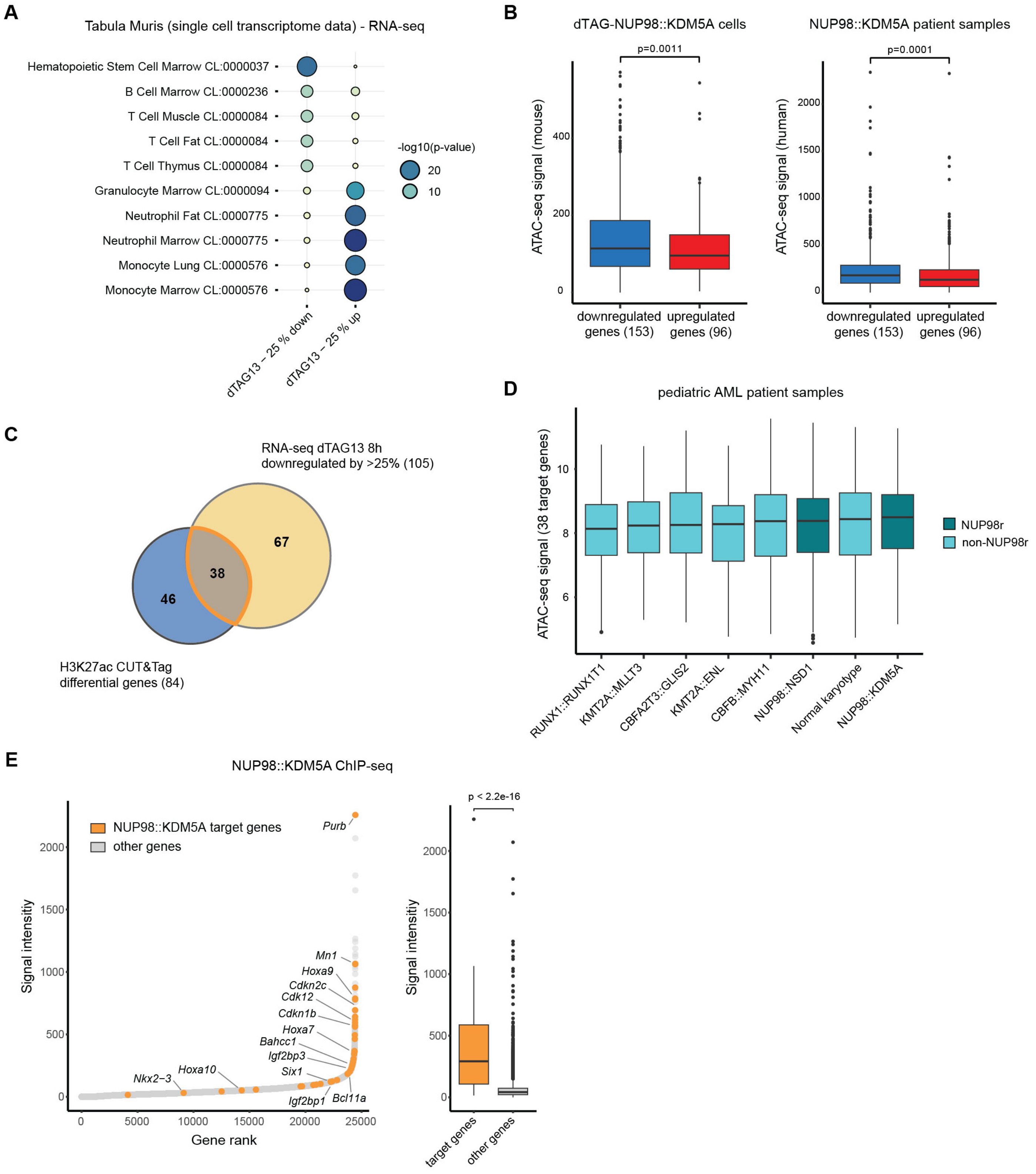

Supplementary Figure 4. NUP98::KDM5A degradation induces global gene expression changes

(A) Gene ontology analysis of RNA-seq data showing top enriched terms of the 25% up- and downregulated genes after dTAG13 treatment compared to DMSO (Tabula Muris). **(B)** ATAC-seq signal of regions associated with 153 downregulated and 96 upregulated genes after 24h dTAG13 treatment in dTAG-NUP98::KDM5A cells and NUP98::KDM5A patient samples (Wilcoxon rank sum test). **(C)** Intersection of H3K27ac CUT&Tag data and RNA-seq data showing the overlap of genes with differential H3K27ac signals and the 25% downregulated genes after dTAG13 treatment compared to DMSO, revealing 38 NUP98::KDM5A target genes. **(D)** ATAC-seq signal of regions associated with the 38 NUP98::KDM5A target genes in pediatric AML patient samples. **(E)** Hockey stick plot of ChIP-seq data of global NUP98::KDM5A chromatin binding in a HA-NUP98::KDM5A-expressing cell line with anti-HA antibody (left). All genes were ranked according to their normalized read signal, including a quantitative box plot representation (right, Wilcoxon rank sum test with continuity correction). For (B,D,E right): Data are presented as boxplots where the center line represents the median, the bounds of the box indicate the first (25th percentile) and third (75th percentile) quartiles and the whiskers extend to 1.5 * inter-quartile range from the hinges. Data points beyond this range are shown as individual dots and represent outliers. Source data are provided as a Source Data file.

Supplementary Figure 5

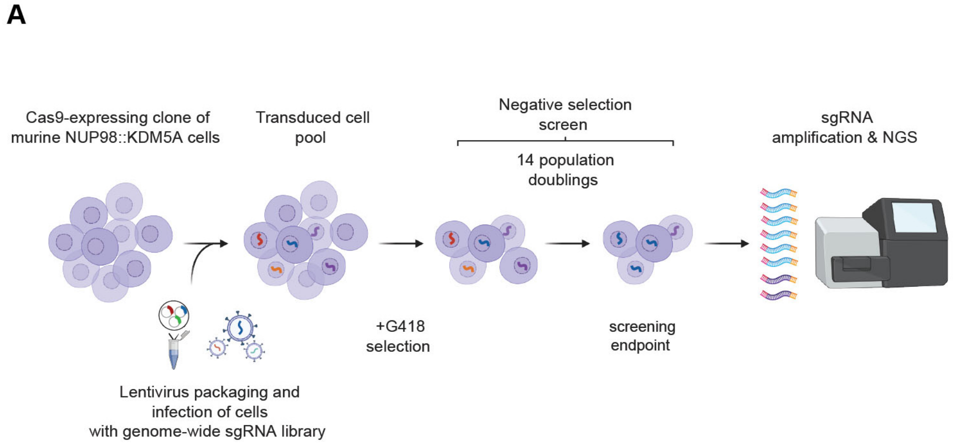

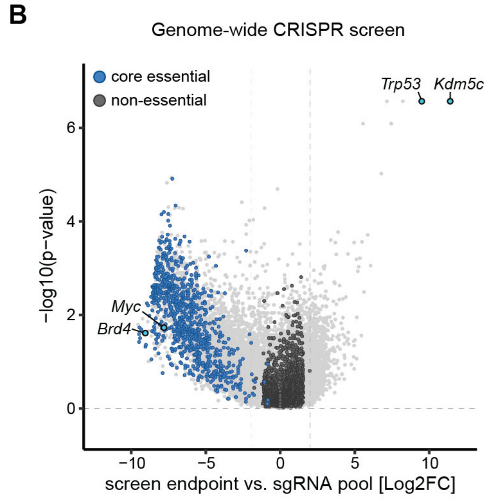

Supplementary Figure 5. Workflow of a genome-wide CRISPR/Cas9 loss-of-function screen in a NUP98::KDM5A-dependent cell line

(A) Schematic overview of the workflow of a genome-wide CRISPR loss-of-function screen in a Cas9-expressing clone of a NUP98::KDM5A-dependent cell line. **(B)** Volcano plot of genome-wide CRISPR/Cas9 screen data showing the log2FC and –log10(p-value) values of mean sgRNA abundance at the screen endpoint vs. the sgRNA pool, highlighting core-essential and non-essential genes (differential regions were identified using the R package DiffBind, applying DESeq2). Parts of the figure were created in BioRender. Grebien, F. (2025). *https://BioRender.com/m67n802*. Source data are provided as a Source Data file.

Supplementary Figure 6

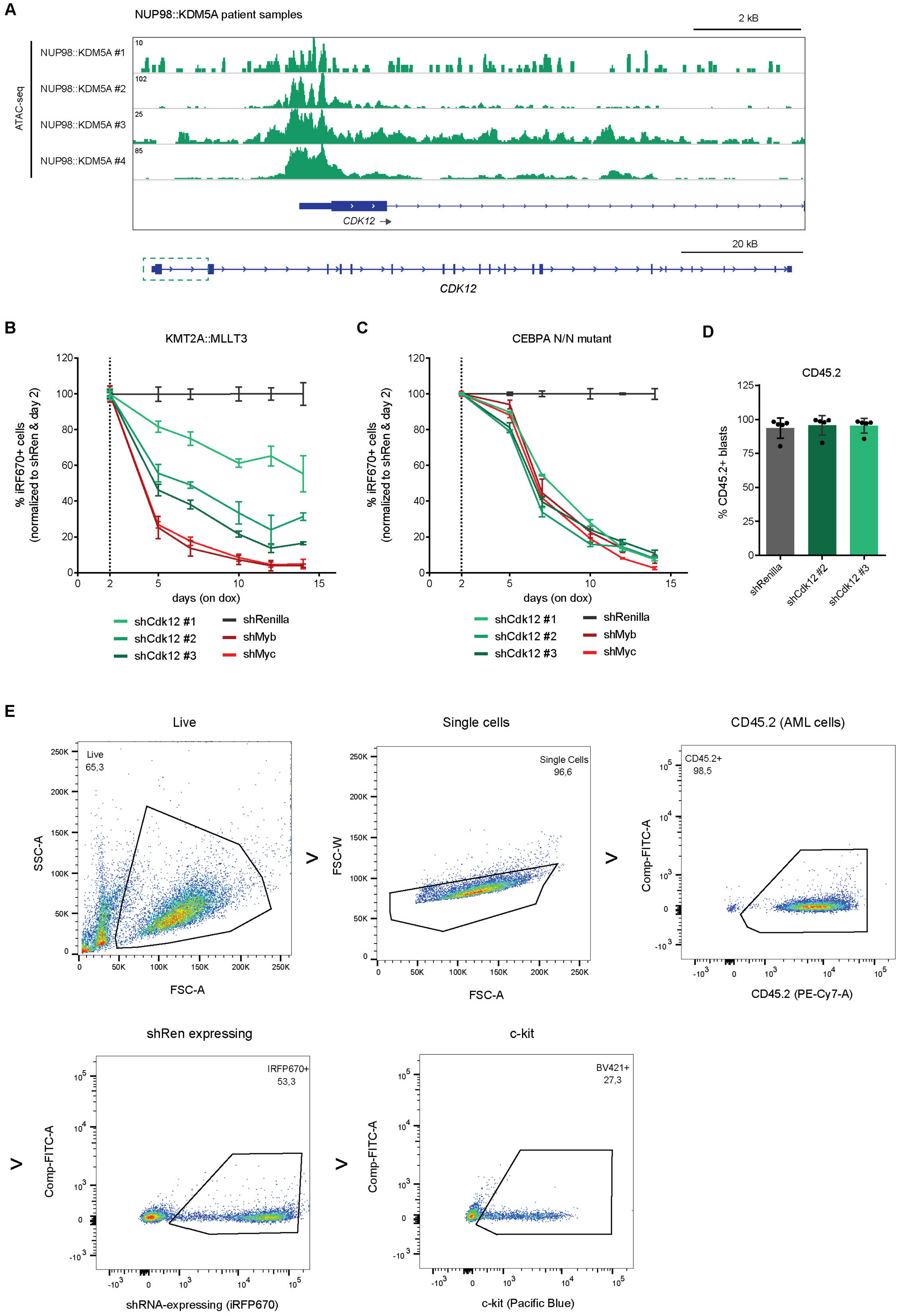

Supplementary Figure 6. CDK12 in AML patient samples and murine AML cell lines

(A) Representative IGV tracks of ATAC-seq data from NUP98::KDM5A patient samples showing the *CDK12* locus. **(B)** Competition-based proliferation assay upon doxycycline-inducible shRNA-mediated knockdown of *Cdk12* in a murine KMT2A::MLLT3 AML cell line **(C)** and a murine N-terminal CEBPA mutant (*Cebpa*$^{p30/p30}$) AML cell line, showing percentage of iRFP670-positive cells over 14 days (n = 3). **(D)** Flow cytometric analysis of BM of recipient mice showing the percentage of CD45.2 surface marker expression (n = 5, mean ± SD). **(E)** Gating strategy for plots shown in Figure 6G. Source data are provided as a Source Data file.

Supplementary Figure 7

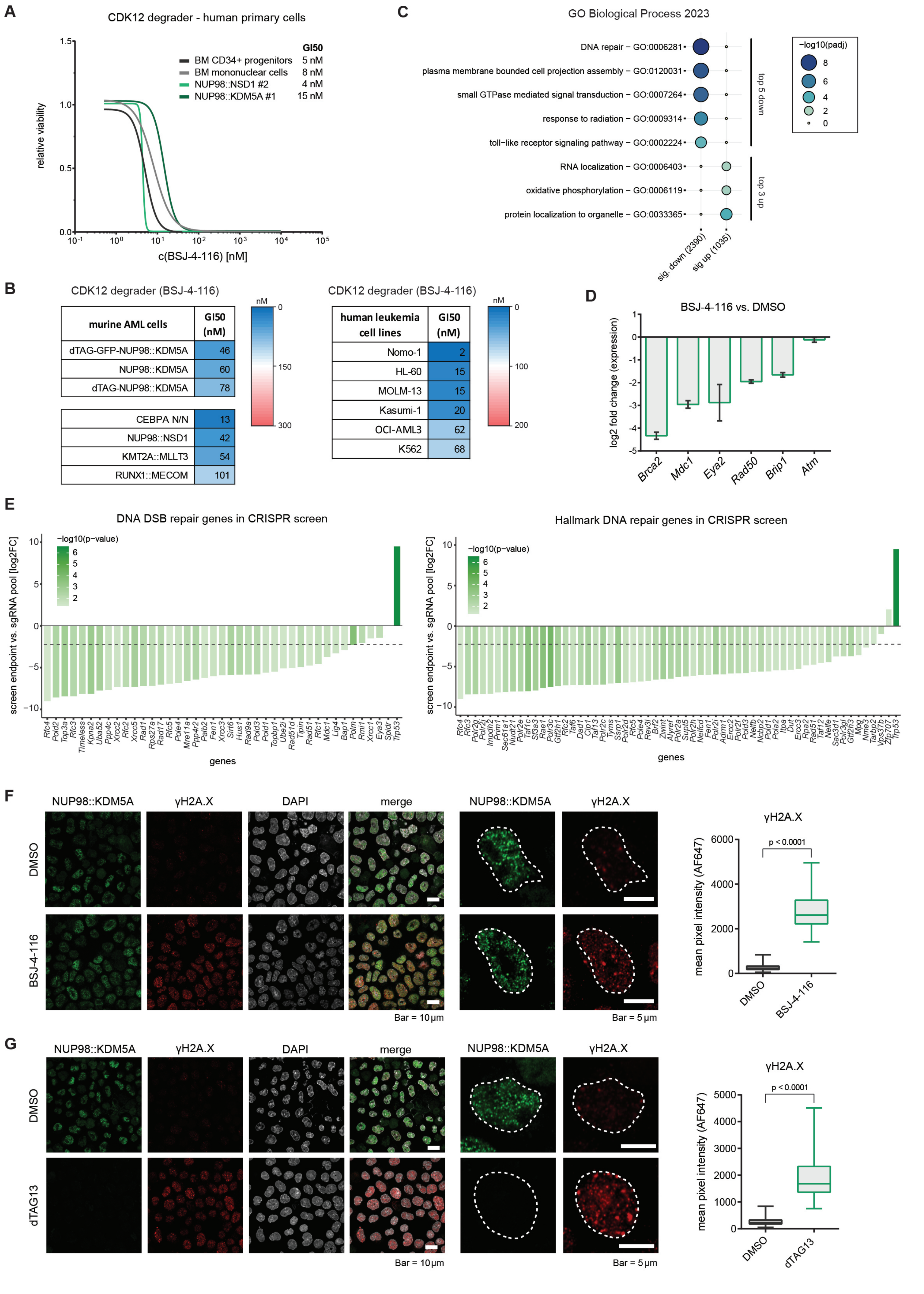

Supplementary Figure 7. CDK12 dependency in NUP98::KDM5A-driven leukemia involves DNA repair

(A) Viability assay of primary human NUP98-rearranged AML cells, healthy donor BM MNC and CD34+ progenitors treated with indicated concentrations of BSJ-4-116 for 3 days. **(B)** GI50 values from cell viability assays of murine AML cells and human leukemia cell lines treated with BSJ-4-116 for 3 days. **(C)** Gene ontology analysis of BSJ-4-116 vs. DMSO RNA-seq data (GO Biological Process 2023). **(D)** RNA-seq data of dTAG-NUP98::KDM5A cells treated with BSJ-4-116 (100 nM, 4h) compared to DMSO showing log2(FC) values of selected DNA damage repair genes (n = 4, mean ± SD). **(E)** Data from the NUP98::KDM5A genome-wide CRISPR screen showing genes of the hallmark DNA repair gene set and genes of the Reactome DNA double strand break repair gene set with significant log2FC values ($p < 0.05$). **(F)** Representative images of γH2A.X immunofluorescence staining of dTAG-GFP-NUP98::KDM5A cells treated with BSJ-4-116 (100 nM, 4h) Representative images of n = 8 samples. **(G)** or dTAG13 (35 nM, 72h), and quantification of γH2A.x signal. Midline represents the median, bounds of the box represent the interquartile range (Q1 to Q3), and whiskers represent minimum and maximum values. (DMSO: n = 125 nuclei (technical replicates), dTAG13: n = 134 nuclei (technical replicates), unpaired t-test, right). Representative images of n = 8 samples. Source data are provided as a Source Data file.

Supplementary Figure 8

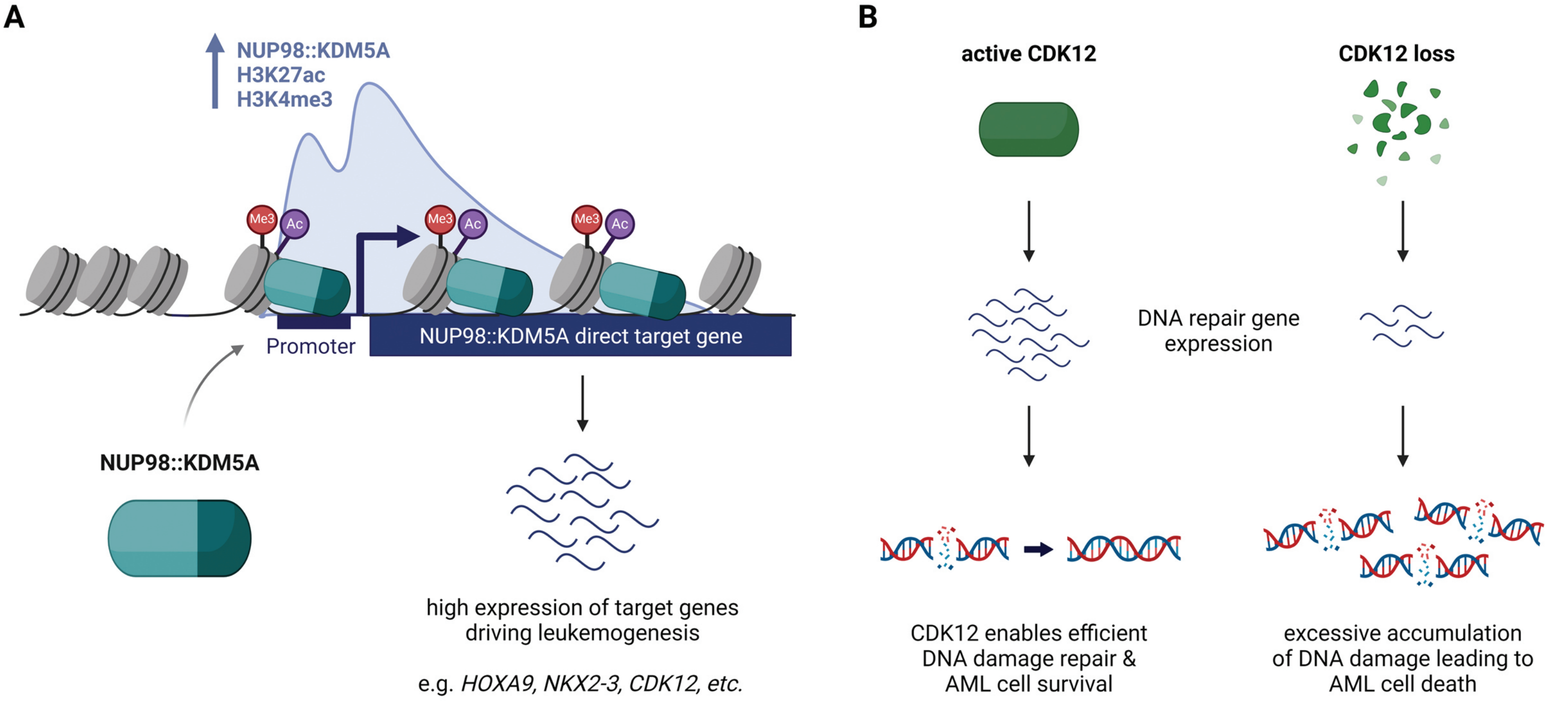

Supplementary Figure 8. Transcriptional and epigenetic rewiring by the NUP98::KDM5A fusion oncoprotein directly activates CDK12 and involves DNA repair

(A) Direct target genes of NUP98::KDM5A are bound by the fusion oncoprotein and are marked by activating H3K27ac and H3K4me3 histone modifications, thereby maintaining high expression of target genes driving leukemogenesis. **(B)** The direct NUP98::KDM5A target CDK12 enables efficient transcription of DNA repair genes and thus prevents excessive accumulation of DNA damage that might occur due to oncogene-induced replication stress, which is a described source of genomic instability. Parts of the figure were created in BioRender. Grebien, F. (2025) *https://BioRender.com/c87k575*.

Uncropped scans of blots in Supplementary Figure 2H and Supplementary Figure 3B

NRAS

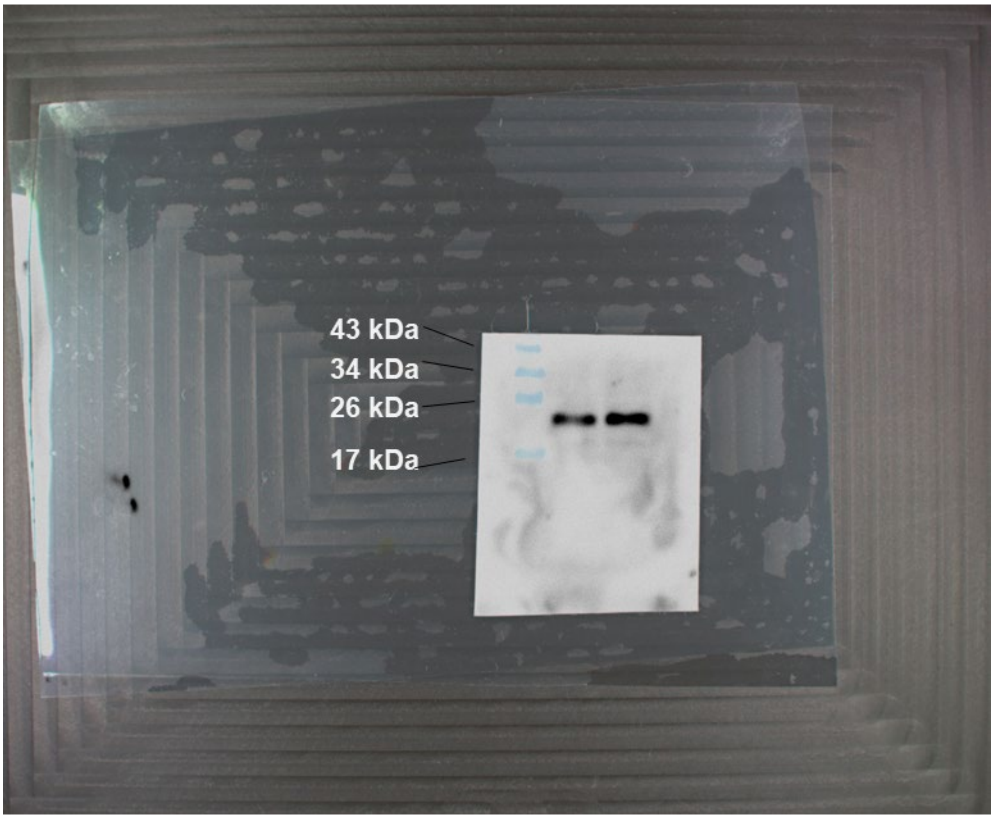

H3 and HSC70

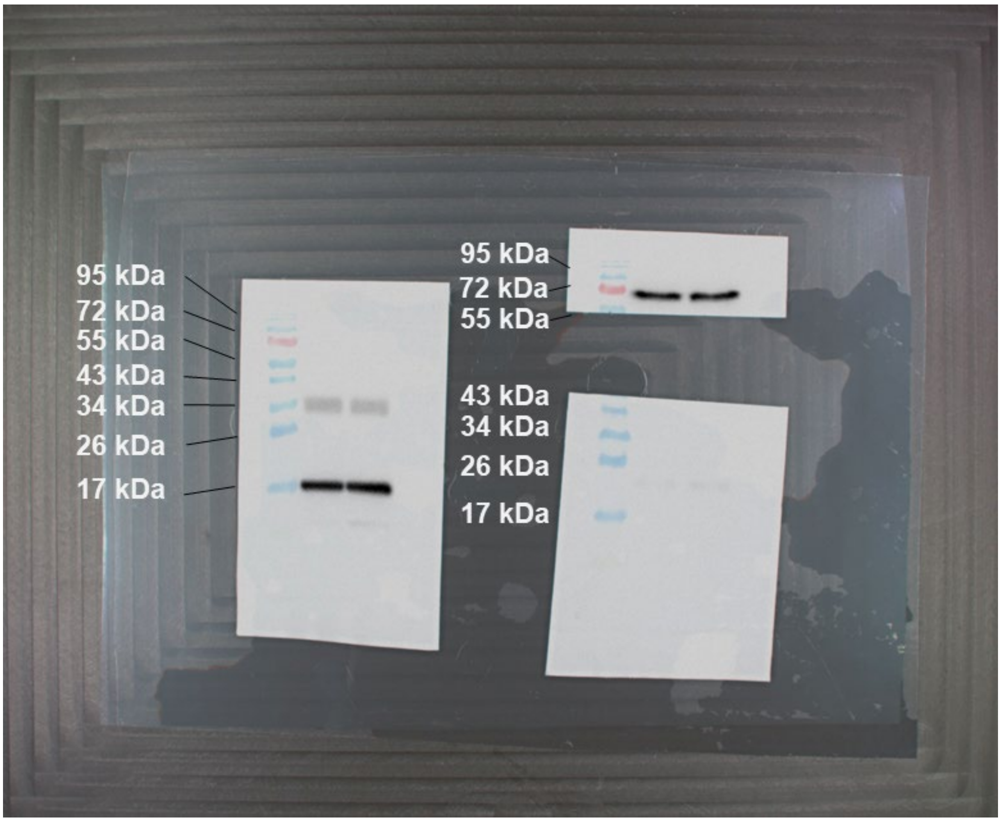

H3K27ac

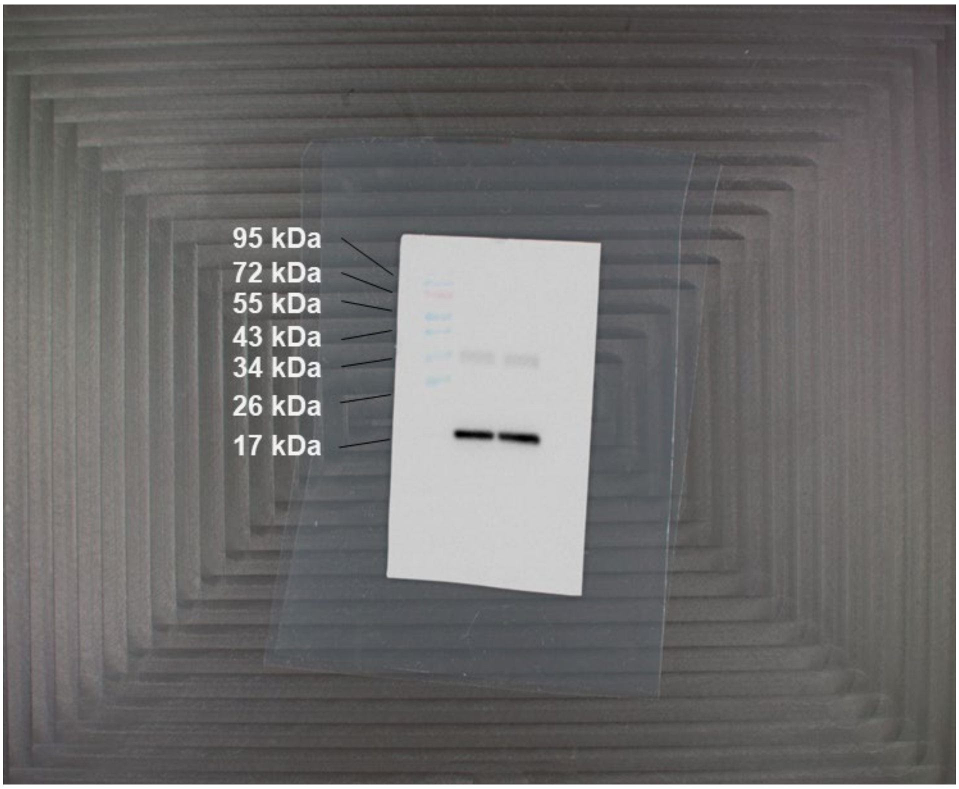

H3K4me3

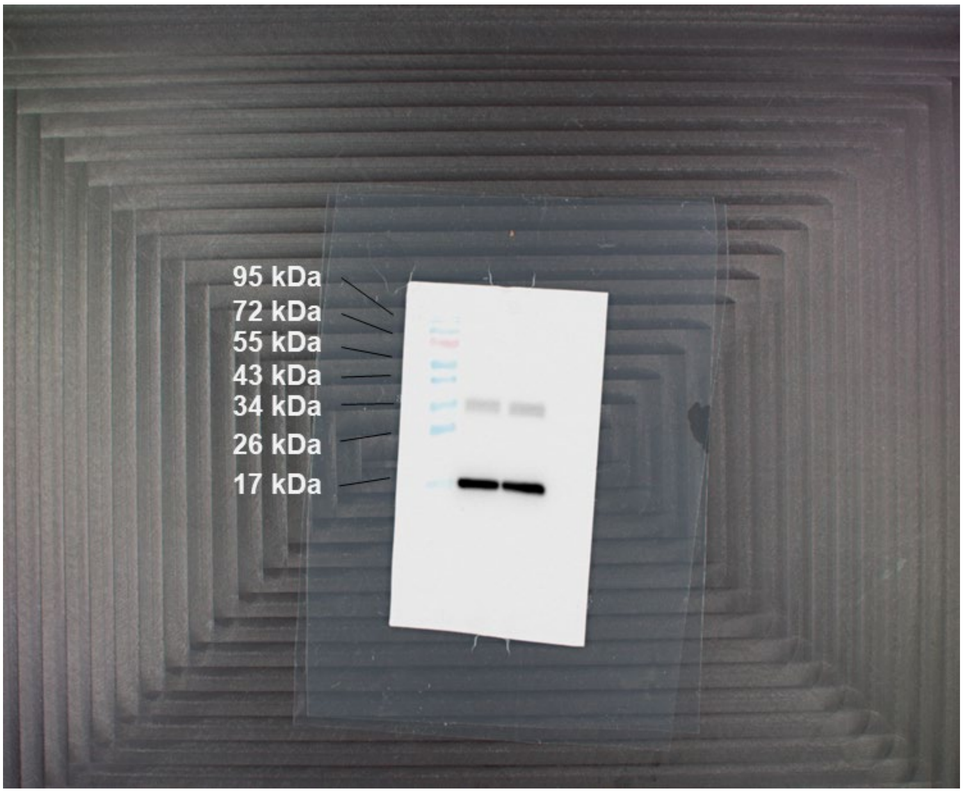

Supplement: Supplementary file 1 — Supplementary Information [file 41467_2025_59930_MOESM1_ESM.pdf]
