# Supplementary material for: Transcriptional and epigenetic rewiring by the NUP98::KDM5A fusion oncoprotein directly activates CDK12
Source: Nat Commun. 2025 May 19;16:4656. doi: 10.1038/s41467-025-59930-9 (PMC12089343; doi:10.1038/s41467-025-59930-9)
Supplement: Supplementary file 9 — Reporting Summary [file 41467_2025_59930_MOESM9_ESM.pdf]

nature portfolio

Corresponding author(s): Florian Grebien

Last updated by author(s): Jan 29, 2025

Reporting Summary

Nature Portfolio wishes to improve the reproducibility of the work that we publish. This form provides structure for consistency and transparency in reporting. For further information on Nature Portfolio policies, see our Editorial Policies and the Editorial Policy Checklist.

Statistics

For all statistical analyses, confirm that the following items are present in the figure legend, table legend, main text, or Methods section.

n/a	Confirmed	
[ ]	[x]	The exact sample size (*n*) for each experimental group/condition, given as a discrete number and unit of measurement
[ ]	[x]	A statement on whether measurements were taken from distinct samples or whether the same sample was measured repeatedly
[ ]	[x]	The statistical test(s) used AND whether they are one- or two-sided<br>*Only common tests should be described solely by name; describe more complex techniques in the Methods section.*
[x]	[ ]	A description of all covariates tested
[ ]	[x]	A description of any assumptions or corrections, such as tests of normality and adjustment for multiple comparisons
[ ]	[x]	A full description of the statistical parameters including central tendency (e.g. means) or other basic estimates (e.g. regression coefficient) AND variation (e.g. standard deviation) or associated estimates of uncertainty (e.g. confidence intervals)
[ ]	[x]	For null hypothesis testing, the test statistic (e.g. *F*, *t*, *r*) with confidence intervals, effect sizes, degrees of freedom and *P* value noted<br>*Give P values as exact values whenever suitable.*
[x]	[ ]	For Bayesian analysis, information on the choice of priors and Markov chain Monte Carlo settings
[x]	[ ]	For hierarchical and complex designs, identification of the appropriate level for tests and full reporting of outcomes
[ ]	[x]	Estimates of effect sizes (e.g. Cohen's *d*, Pearson's *r*), indicating how they were calculated

Our web collection on statistics for biologists contains articles on many of the points above.

Software and code

Policy information about availability of computer code

Data collection	
Data collection	Genome wide CRISPR screen data was obtained with an Illumina HiSeqV4, nascent RNA-seq data with an Illumina NovaSeq SP and RNA-seq and CUT&Tag data with an Illumina NextSeq2000 machine.<br><br>Whole-body fluorescence imaging was performed with an IVIS optical imaging system (PerkinElmer, USA) using Living Image software.<br><br>Flow cytometry: Samples were measured either with a FACS Canto II flow cytometer (BD Biosciences, Germany) using BD FACSDiva Software (v7.0) or an IntelliCyt IQueScreener Plus (BioScience, Sartorius Group, Germany) using Forecyt standard Edition 7.0 (R2) (7.0.7035).<br><br>Cell viability: Determined using the CellTiter-Glo® Luminescent Cell Viability Assay (Promega, USA), on a Spark multimode microplate reader (TECAN, Switzerland) using SparkControl software (v2.3)<br><br>Cytospins: Photographs were taken on a Zeiss Imager Z.1 microscope and images were processed using Zeiss ZEN software (Zeiss, Jena, Germany).<br><br>Immunofluorescence microscopy: Images were acquired using an inverted confocal microscope with Airyscan super-resolution capability Zeiss LSM 880 Airyscan (Zeiss, Jena, Germany) and a 63x oil objective (Zeiss 63x/1.40 Plan-Apochr., Oil, DIC III) in Airyscan mode and Zen Black software.<br><br>Real-time PCR analysis were performed on a Bio-Rad CFX96-Real-Time PCR Detection System.

Data analysis

Bioinformatic analysis of the genome wide CRISPR screen:
Raw .bam files underwent processing using the crispr-process-nf Nextflow pipeline, available at https://github.com/ZuberLab/crispr-process-nf, which included barcode trimming, filtering, and alignment using Bowtie2. The crispr-mageck-nf Nextflow pipeline, available at https://github.com/ZuberLab/crispr-mageck-nf incorporating MAGeCK, was employed for comprehensive analysis of the CRISPR screen data.

Bioinformatic analysis of the RNA-seq data:
Raw files underwent preprocessing using prinseq-lite (version 0.20.4). Subsequently, alignment against the mouse reference genome (mm10) was performed using STAR (version 2.7.9a). Post-processing and sorting of aligned reads was conducted using samtools (version 1.4). Counts per gene were obtained utilizing featureCounts (version 2.0.3) from the subread package. Normalized expression levels and identification of differentially expressed genes were calculated using the DESeq2 R package. Heatmaps depicting expression patterns were created using the heatmap.2 function from the gplots R package. Time-series plots illustrating dynamic expression changes over time were generated using the maSigPro R library. Gene set enrichment analysis was performed with GSEA software (version 4.3.2). GO-enrichment was performed with EnrichR and g:Profiler (g:GOst) and plots were generated with SR-plot.

Bioinformatic analysis of the nascent RNA-seq data:
Raw .bam files underwent processing using the slamseq Nextflow pipeline, accessible at https://github.com/nf-core/slamseq. This pipeline integrates multiple tools including fastqc, trim_galore and slamdunk available at: https://github.com/t-neumann/slamdunk, for quality assessment, adapter trimming, and read mapping. DESeq2 was employed for comparative analysis, facilitating the identification of newly transcribed genes. Heatmaps depicting expression patterns were created using the heatmap.2 function from the gplots R package.

Bioinformatic analysis of the CUT&Tag data:
Raw files underwent preprocessing using prinseq-lite for quality control and filtering. Alignment against either the mouse (mm10) or human (hg38) reference genomes was conducted using BWA (version 0.7.17-r1188). Post-processing and sorting of aligned reads were performed using samtools (version 1.13). The function bamCoverage from Deeptools (version 3.5.1) was utilized to generate BigWig files with a binSize of 10, employing Counts Per Million (CPM) for normalization. Peaks were called using macs2 (version 2.1.0) with the --broad option. Differential CUT&Tag regions were identified using the R package DiffBind, applying DESeq2. Tornado and profile plots were created using the generateEnrichedHeatmap from the profileplyr R package or Deeptools. Read counts per genes were obtained using featureCounts from the subread package. All hockey-stick and scatter plots were generated using ggplot2 in R. Coverage plots were generated with IGV.

Bioinformatic analysis of the ATAC-seq data:
Publicly available AML patient ATAC-seq datasets were obtained from GEO accessions listed in supplementary table 1. Pediatric AML patient data were obtained from the St. Anna Children's Cancer Research Institute (CCRI) and Yokohama City University Hospital.
A modified version of the ATAC-seq Data Processing Pipeline was applied to the raw Bam files, accessible at: https://github.com/epigen/atacseq_pipeline. The pipeline utilized fastp for adapter removal and Bowtie2 for read alignment to the GRCh38 (hg38) human reference genome. Duplicate marking was performed with samblaster and aligned BAM files were sorted, indexed, and filtered for ENCODE blacklisted regions using samtools. Peaks were called with macs2 (version 2.1.0) applying the --broad option. Differential ATAC-seq regions were identified with DiffBind and region-to-gene annotation was conducted with ChIPseeker. Counts over exons were obtained using featureCounts and normalization was performed with DESeq2. Principal Component Analysis (PCA) plots were generated using the ggplot2 package in R. For the minimum spanning tree visualization batch correction was done with ComBat and we utilized vegdist from the VEGAN package and ggraph. Tornado and profile plots were created with Deeptools and the differential ATAC-seq regions showcased via heatmaps were generated with DiffBind.

Whole-body fluorescence imaging:
Signal quantification was performed using the Living Image analysis software (PerkinElmer, USA). Living image analysis software Version 4.7.3.

Flow cytometry: Analysis was performed with the FlowJo software v10.8.1 (FlowJo, LLC) or Forecyt standard Edition 7.0 (R2) (7.0.7035).

Immunofluorescence microscopy: Image analysis was performed with Arivis Vision 4D software (Arivis AG, Berlin, Germany).

The Prism 6.0.1 software (Graphpad, USA) and Microsoft Excel 2016 were used for data compilation and for statistical analyses.

For manuscripts utilizing custom algorithms or software that are central to the research but not yet described in published literature, software must be made available to editors and reviewers. We strongly encourage code deposition in a community repository (e.g. GitHub). See the Nature Portfolio guidelines for submitting code & software for further information.

Data

Policy information about availability of data

All manuscripts must include a data availability statement. This statement should provide the following information, where applicable:

- Accession codes, unique identifiers, or web links for publicly available datasets
- A description of any restrictions on data availability
- For clinical datasets or third party data, please ensure that the statement adheres to our policy

The publicly available Tet-Off RNA-seq data used in this study are available in the Gene Expression Omnibus (GEO) database under accession code GSE134784 [https://www.ncbi.nlm.nih.gov/geo/query/acc.cgi?acc=GSE134784].
The raw ATAC-seq data from primary pediatric AML samples obtained from the Yokohama City University (YCU) are protected and are not available due to data privacy laws. The processed ATAC-seq count data are available on Zenodo under DOI 10.5281/zenodo.14943880 [https://10.5281/zenodo.14943880].
The publicly available ATAC-seq data from 15 primary pediatric AML samples obtained from the St. Anna Children's Cancer Research Institute (CCRI) used in this study are available in the GEO database under accession code GSE282258 [https://www.ncbi.nlm.nih.gov/geo/query/acc.cgi?acc=GSE282258]. The mapping of sample names to GEO accessions is provided in Supplementary Data 1.
The genome-wide CRISPR-screen, RNA (QUANT)-seq, nascent (SLAM)-seq, Cut&Tag and ATAC-seq cell line data generated in this study have been deposited in the GEO database under accession code GSE255808 [https://www.ncbi.nlm.nih.gov/geo/query/acc.cgi?acc=GSE255808]

The remaining data are available within the Article, Supplementary Information or Source Data file. Source data are provided with this paper.

Research involving human participants, their data, or biological material

Policy information about studies with human participants or human data. See also policy information about sex, gender (identity/presentation), and sexual orientation and race, ethnicity and racism.

Reporting on sex and gender	In this study, samples from pediatric AML patients were analyzed, including samples from the biobank of the St. Anna Children's Hospital Vienna, Austria and samples that have been published previously by Yamato, G. et al. (https://doi.org/10.1182/bloodadvances.2021005381). In total, NUP98-rearranged samples from female (n = 4) and male (n = 6) patients were analyzed and NUP98 wild-type samples from female (n = 5) and male (n = 5) patients were analyzed. For the in vivo Cdk12 shRNA knockdown experiment, each cohort contained a balanced number of male and female mice.
Reporting on race, ethnicity, or other socially relevant groupings	No socially constructed or socially relevant categorization variables were used in this study.
Population characteristics	For this study we compared ATAC-seq data from pediatric AML patient samples according to their mutational status of the NUP98 gene: NUP98-rearranged vs. NUP98 wild-type.
Recruitment	Samples were collected at diagnosis from patients enrolled in the AML-BFM studies. All patients or their respective legal guardians gave written informed consent prior to the study. Samples that were used in this study were selected based on mutational status of the NUP98 gene. In addition to NUP98-rearranged patient samples, NUP98 wild-type AML patient samples with other oncogenic drivers were included in this study for comparison.
Ethics oversight	Fresh-frozen samples of primary bone-marrow mononuclear cells (MNCs) were obtained from the biobank of the St. Anna Children's Hospital Vienna, Austria. Collection of samples and their use in research was performed with clearance of the appropriate Ethics committee (ethics vote No.1500/2014 of the Ethics Committee of the Medical University Vienna, Vienna, Austria).

Note that full information on the approval of the study protocol must also be provided in the manuscript.

Field-specific reporting

Please select the one below that is the best fit for your research. If you are not sure, read the appropriate sections before making your selection.

[x] Life sciences [ ] Behavioural & social sciences [ ] Ecological, evolutionary & environmental sciences

For a reference copy of the document with all sections, see nature.com/documents/nr-reporting-summary-flat.pdf

Life sciences study design

All studies must disclose on these points even when the disclosure is negative.

Sample size	An appropriate sample size was chosen based on the magnitude and consistency of measurable differences between groups, as well as based on availability and practical reasons and costs. All sample sizes are indicated in the figure legends.
Data exclusions	In Fig. 1C and Fig. 1D ATAC-seq signals of patient samples "NUP98::KDM5A #2/3/4" are plotted. We excluded patient sample "NUP98::KDM5A #1" because it substanially differed from the other three consistent NUP98::KDM5A patient samples, as can be seen in Fig. 1E. Otherwise, no data was excluded.
Replication	Most experiments were replicated at least two times, except NGS or patients and animal experiments. NGS analyses with patient samples were not replicated due to inavailability of material. Replication of animal experiments was performed in accordance with 3R guidelines. All attempts at replication were successful.
Randomization	For the in vivo Cdk12 shRNA knockdown experiment, each cohort contained a balanced number of male and female mice. Otherwise, age-matched mice (13-14 weeks old) were randomly distributed into three groups of equal size and transplanted with equal cell numbers of either doxycycline-inducible shRenilla, shCdk12-2 or shCdk12-3 expressing NUP98::KDM5A AML cells. All cohorts were administered with doxycycline (4mg/ml) via the drinking water.<br>Cell culture experiments were not randomized as the experimental groups needed to be known for subsequent analysis.
Blinding	Investigators were not blinded to the group allocation of mice since the kowledge of the genotype of transplanted cells needed to be known for subsequent analysis.

Behavioural & social sciences study design

All studies must disclose on these points even when the disclosure is negative.

Study description	*Briefly describe the study type including whether data are quantitative, qualitative, or mixed-methods (e.g. qualitative cross-sectional, quantitative experimental, mixed-methods case study).*
Research sample	*State the research sample (e.g. Harvard university undergraduates, villagers in rural India) and provide relevant demographic information (e.g. age, sex) and indicate whether the sample is representative. Provide a rationale for the study sample chosen. For studies involving existing datasets, please describe the dataset and source.*
Sampling strategy	*Describe the sampling procedure (e.g. random, snowball, stratified, convenience). Describe the statistical methods that were used to predetermine sample size OR if no sample-size calculation was performed, describe how sample sizes were chosen and provide a rationale for why these sample sizes are sufficient. For qualitative data, please indicate whether data saturation was considered, and what criteria were used to decide that no further sampling was needed.*
Data collection	*Provide details about the data collection procedure, including the instruments or devices used to record the data (e.g. pen and paper, computer, eye tracker, video or audio equipment) whether anyone was present besides the participant(s) and the researcher, and whether the researcher was blind to experimental condition and/or the study hypothesis during data collection.*
Timing	*Indicate the start and stop dates of data collection. If there is a gap between collection periods, state the dates for each sample cohort.*
Data exclusions	*If no data were excluded from the analyses, state so OR if data were excluded, provide the exact number of exclusions and the rationale behind them, indicating whether exclusion criteria were pre-established.*
Non-participation	*State how many participants dropped out/declined participation and the reason(s) given OR provide response rate OR state that no participants dropped out/declined participation.*
Randomization	*If participants were not allocated into experimental groups, state so OR describe how participants were allocated to groups, and if allocation was not random, describe how covariates were controlled.*

Ecological, evolutionary & environmental sciences study design

All studies must disclose on these points even when the disclosure is negative.

Study description	*Briefly describe the study. For quantitative data include treatment factors and interactions, design structure (e.g. factorial, nested, hierarchical), nature and number of experimental units and replicates.*
Research sample	*Describe the research sample (e.g. a group of tagged Passer domesticus, all Stenocereus thurberi within Organ Pipe Cactus National Monument), and provide a rationale for the sample choice. When relevant, describe the organism taxa, source, sex, age range and any manipulations. State what population the sample is meant to represent when applicable. For studies involving existing datasets, describe the data and its source.*
Sampling strategy	*Note the sampling procedure. Describe the statistical methods that were used to predetermine sample size OR if no sample-size calculation was performed, describe how sample sizes were chosen and provide a rationale for why these sample sizes are sufficient.*
Data collection	*Describe the data collection procedure, including who recorded the data and how.*
Timing and spatial scale	*Indicate the start and stop dates of data collection, noting the frequency and periodicity of sampling and providing a rationale for these choices. If there is a gap between collection periods, state the dates for each sample cohort. Specify the spatial scale from which the data are taken*
Data exclusions	*If no data were excluded from the analyses, state so OR if data were excluded, describe the exclusions and the rationale behind them, indicating whether exclusion criteria were pre-established.*
Reproducibility	*Describe the measures taken to verify the reproducibility of experimental findings. For each experiment, note whether any attempts to repeat the experiment failed OR state that all attempts to repeat the experiment were successful.*
Randomization	*Describe how samples/organisms/participants were allocated into groups. If allocation was not random, describe how covariates were controlled. If this is not relevant to your study, explain why.*
Blinding	*Describe the extent of blinding used during data acquisition and analysis. If blinding was not possible, describe why OR explain why blinding was not relevant to your study.*

Did the study involve field work? ☐ Yes ☐ No

Field work, collection and transport

Field conditions	*Describe the study conditions for field work, providing relevant parameters (e.g. temperature, rainfall).*
Location	*State the location of the sampling or experiment, providing relevant parameters (e.g. latitude and longitude, elevation, water depth).*
Access & import/export	*Describe the efforts you have made to access habitats and to collect and import/export your samples in a responsible manner and in compliance with local, national and international laws, noting any permits that were obtained (give the name of the issuing authority, the date of issue, and any identifying information).*
Disturbance	*Describe any disturbance caused by the study and how it was minimized.*

Reporting for specific materials, systems and methods

We require information from authors about some types of materials, experimental systems and methods used in many studies. Here, indicate whether each material, system or method listed is relevant to your study. If you are not sure if a list item applies to your research, read the appropriate section before selecting a response.

Materials & experimental systems

n/a	Involved in the study
☐	☒ Antibodies
☐	☒ Eukaryotic cell lines
☒	☐ Palaeontology and archaeology
☐	☒ Animals and other organisms
☒	☐ Clinical data
☒	☐ Dual use research of concern
☒	☐ Plants

Methods

n/a	Involved in the study
☒	☐ ChIP-seq
☐	☒ Flow cytometry
☒	☐ MRI-based neuroimaging

Antibodies

Antibodies used

Information provided in following order: antigen, name, supplier, catalog number, clone, lot number

Flow cytometry:
CD16/CD32, BD Pharmingen™ Purified Rat Anti-Mouse CD16/CD32 (Mouse BD Fc Block™), BD Biosiences, 553142, 2.4G2, 8243696, 1:200
V5-Tag, V5-Tag Rabbit mAb, Cell signaling, 13202S, D3H8Q, 7, 1:400
CD11b, PerCP/Cyanine5.5 anti-mouse/human CD11b Antibody, Biolegend, 101227, M1/70, B404844, 1:200
Ly-6G/Ly-6C, Brilliant Violet 421™ anti-mouse Ly-6G/Ly-6C (Gr-1) Antibody, Biolegend, 108433, RB6-8C5, B318826, 1:200
Ly-6G/Ly-6C, PE/Cyanine7 anti-mouse Ly-6G/Ly-6C (Gr-1) Antibody,, Biolegend, 108416, RB6-8C5, B284962, 1:200
CD117, APC anti-mouse CD117 (c-Kit) Antibody,, Biolegend, 105812, 2B8, B217855, 1:200
Rabbit IgG, F(ab')2-Goat anti-Rabbit IgG (H+L) Cross-Adsorbed Secondary Antibody Alexa Fluor™ 647, Thermo Fisher Scientific, A-21244, polyclonal, 913922, 1:200
CD45.2, PE/Cyanine7 anti-mouse CD45.2 Antibody, Biolegend, 109829, 104, 1:200
c-KIT, Biotin anti-mouse CD117 (c-Kit) Antibody, Biolegend, 105803, 2B8, 1:200

Western blot:
H3K4me3, Anti-Histone H3 (tri methyl K4) antibody - ChIP Grade, Abcam, ab8580, polyclonal, GR3190162-1, 1:1000
H3K27ac, Anti-Histone H3 (acetyl K27) antibody - ChIP Grade, Abcam, ab4729, polyclonal, GR3187597-1, 1:1000
Histone H3, Anti-Histone H3 antibody - Nuclear Marker and ChIP Grade, Abcam, ab1791, polyclonal, GR3198209-1, 1:1000
HSC70, HSPA8/HSC70 antibody, Santa Cruz, sc-7298, monoclonal, B-6, B2117, 1:10 000
Nras, N-Ras antibody, Santa Cruz, sc-31, monoclonal, F155, 1:1000

ChIP-qPCR:
H3K4me3, Anti-Histone H3 (tri methyl K4) antibody - ChIP Grade, Abcam, ab8580, polyclonal, GR3190162-1, 1 µg/µl
H3K27ac, Anti-Histone H3 (acetyl K27) antibody - ChIP Grade, Abcam, ab4729, polyclonal, GR3187597-1, 1 µg/µl

CUT&Tag:
H3K4me3, Anti-Histone H3 (tri methyl K4) antibody - ChIP Grade, Abcam, ab8580, polyclonal, GR3190162-1, 1 µg
H3K27ac, Anti-Histone H3 (acetyl K27) antibody - ChIP Grade, Abcam, ab4729, polyclonal, GR3187597-1, 1 µg
Rabbit IgG, Rabbit IgG, Diagenode, C15410206, Polyclonal, RIG002B, 1 µg
Rabbit IgG, Rabbit IgG, Diagenode, C01070022, N/A, RIG001 (included in Antibody package for CUT&Tag (anti-rabbit)), 1 µg
Anti-rabbit secondary antibody, Diagenode, Belgium C01070022, N/A, P001 (included in Antibody package for CUT&Tag (anti-rabbit)),

1:100

Immunofluorescence staining:
gamma H2A.X, Anti-gamma H2A.X (phospho S139) antibody, Abcam, ab2893, polyclonal, GR3223242-1, 1:1500
Rabbit IgG, F(ab')2-Goat anti-Rabbit IgG (H+L) Cross-Adsorbed Secondary Antibody Alexa Fluor™ 647, Thermo Fisher Scientific, A-21244, polyclonal, 913922, 1:1500

Validation

BD Pharmingen™ Purified Rat Anti-Mouse CD16/CD32 (Mouse BD Fc Block™): The 2.4G2 antibody specifically recognizes a common nonpolymorphic epitope on the extracellular domains of the mouse FcγIII (CD16) and FcγII (CD32) Receptors. It has also been reported to bind the FcγI receptor (CD64) via its Fc domain. 2.4G2 mAb blocks non-antigen-specific binding of immunoglobulins to the FcγIII and FcγII, and possibly FcγI, Receptors in vitro and in vivo. Reactivity: Mouse (QC Testing). Citations: 19, most recent: 10.1042/0300-5127:0290840

V5-Tag Rabbit mAb: V5-Tag (D3H8Q) Rabbit mAb recognizes transfected levels of recombinant protein containing the V5 epitope tag. Species Reactivity: All Species Expected. Citations: 369, most recent: 10.1038/s41467-023-43657-6

F(ab')2-Goat anti-Rabbit IgG (H+L) Cross-Adsorbed Secondary Antibody, Alexa Fluor™ 647: This Antibody was verified by Relative expression to ensure that the antibody binds to the antigen stated. Citations: 1452, most recent: 10.1038/s41467-024-45760-8

PerCP/Cyanine5.5 anti-mouse/human CD11b Antibody: Each lot of this antibody is quality control tested by immunofluorescent staining with flow cytometric analysis. Verified Reactivity: Mouse, Human, Cynomolgus, Rhesus. Citations: 239, most recent: 10.1038/s41467-022-31993-y

Brilliant Violet 421™ anti-mouse Ly-6G/Ly-6C (Gr-1) Antibody: Each lot of this antibody is quality control tested by immunofluorescent staining with flow cytometric analysis. Verified Reactivity: Mouse. Citations: 21, most recent: 10.1016/j.crmeth.2022.100315

APC anti-mouse CD117 (c-Kit) Antibody:Each lot of this antibody is quality control tested by immunofluorescent staining with flow cytometric analysis. Verified Reactivity: Mouse. Citations: 105, most recent: 10.1186/s40164-022-00329-3

PE/Cyanine7 anti-mouse Ly-6G/Ly-6C (Gr-1): Each lot of this antibody is quality control tested by immunofluorescent staining with flow cytometric analysis. Verified Reactivity: Mouse. Citations: 63, most recent: 10.1038/s41467-022-33349-y

Anti-gamma H2A.X (phospho S139) antibody: Validated in ICC/IF, WB and tested in Human, Mouse, Rat samples. Cited in 307 publications.

Anti-Histone H3 (tri methyl K4) antibody - ChIP Grade: Validated in ChIP, WB, PepArr, IHC-P, ICC/IF and tested in Human, Cow, Synthetic peptide - Human samples; predicted: mouse. Cited in 1671 publications.

Anti-Histone H3 (acetyl K27) antibody - ChIP Grade: Validated in IHC-P, ChIP, WB, PepArr, ICC/IF and tested in Human, Rat, Cow, Mouse, Synthetic peptide - Human samples. Cited in 1443 publications.

Rabbit IgG: The negative Ctrl IgG from rabbit has been extensively validated in chromatin immunoprecipitation assays (ChIP). It contains a spectrum of the IgG subclasses present in serum of healthy animals. The negative Ctrl IgG is intended for use as a negative control in ChIP, CUT&Tag, MeDIP, IF and other experiments performed with specific antibodies made in rabbit. Most recent citation: 10.1158/1541-7786.MCR-22-0916

Eukaryotic cell lines

Policy information about cell lines and Sex and Gender in Research

Cell line source(s)

Cells for establishment of the NUP98::KDM5A patient-derived xenograft model were derived from a female patient. Primary patient cells used for drug sensitivity assay in Fig. 6D were derived from a male patient. All murine cell line models were established from murine fetal liver cells and sex was not determined after establishment of the cell lines.

Human cell lines were obtained from DSMZ: Nomo-1 (ACC 542), HL-60 (ACC 3), MOLM-13 (ACC 554), Kasumi-1 (ACC 220), OCI-AML3 (ACC 582) and K562 (ACC 10)

Platinum-E (Cell Biolabs, San Diego, USA)
Lenti-X 293T (Takara, Paris, France)

Authentication

Human cell lines were validated using STR profiling. Primary cell lines were not authenticated, since testing is not applicable for primary samples and primary cell lines.

Mycoplasma contamination

All cell lines were routinely tested for mycoplasma contamination and confirmed negative.

Commonly misidentified lines
(See ICLAC register)

No commonly misidentified cell lines were used.

Palaeontology and Archaeology

Specimen provenance	*Provide provenance information for specimens and describe permits that were obtained for the work (including the name of the issuing authority, the date of issue, and any identifying information). Permits should encompass collection and, where applicable, export.*
Specimen deposition	*Indicate where the specimens have been deposited to permit free access by other researchers.*
Dating methods	*If new dates are provided, describe how they were obtained (e.g. collection, storage, sample pretreatment and measurement), where they were obtained (i.e. lab name), the calibration program and the protocol for quality assurance OR state that no new dates are provided.*

☐ Tick this box to confirm that the raw and calibrated dates are available in the paper or in Supplementary Information.

Ethics oversight	*Identify the organization(s) that approved or provided guidance on the study protocol, OR state that no ethical approval or guidance was required and explain why not.*

Note that full information on the approval of the study protocol must also be provided in the manuscript.

Animals and other research organisms

Policy information about studies involving animals; ARRIVE guidelines recommended for reporting animal research, and Sex and Gender in Research

Laboratory animals	For establishment of murine AML cell lines, fetal liver cells were transduced with oncogene-expressing plasmids and transplanted into recipient mice. For this, male and female C57BL/6J.SJL mice at the age of 10-12 weeks were used. For the characterization of the dTAG-NUP98::KDM5A in vivo model (Supplementary Fig. 2) secondary transplants were performed with female C57BL/6J.SJL mice (n=6) at the age of 10-12 weeks. For in vivo shRNA-induced knockdown of Cdk12 (Fig. 6) murine NUP98::KDM5A AML cells with shRNA-expressing plasmids were transplanted into recipient mice. For this, male and female C57BL/6J.SJL mice (n=15) at the age of 13-14 weeks were used. Mice were kept in specific opportunistic pathogen free quality (SOPF) under stringent controlled standard conditions, in individually ventilated cages, fed with Sniff Haltungsfutter CHOW standard 10mm pellets (Catalog-No. V1534-000), ad libitum.
Wild animals	No wild animals were used in the study.
Reporting on sex	Male and female recepient mice were used in this study for transplantation and establishment of murine AML models. For the in vivo Cdk12 shRNA knockdown experiment, each cohort contained a balanced number of male and female mice.
Field-collected samples	No field collected samples were used in the study.
Ethics oversight	All animal studies were approved by the Ethics and Animal Welfare Committee of the University of Veterinary Medicine, Vienna in accordance with the University's guidelines for Good Scientific Practice and authorized by the Austrian Federal Ministry of Education, Science and Research (ref BMBWF 68.205/0199-V/3b/2018, 2022-0.874.042) in accordance with current legislation.Sex was not considered in the study design because no sex bias for NUP98::KDM5A-driven AML has been reported.

Note that full information on the approval of the study protocol must also be provided in the manuscript.

Clinical data

Policy information about clinical studies

All manuscripts should comply with the ICMJEguidelines for publication of clinical research and a completedCONSORT checklist must be included with all submissions.

Clinical trial registration	*Provide the trial registration number from ClinicalTrials.gov or an equivalent agency.*
Study protocol	*Note where the full trial protocol can be accessed OR if not available, explain why.*
Data collection	*Describe the settings and locales of data collection, noting the time periods of recruitment and data collection.*
Outcomes	*Describe how you pre-defined primary and secondary outcome measures and how you assessed these measures.*

Dual use research of concern

Policy information about dual use research of concern

Hazards

Could the accidental, deliberate or reckless misuse of agents or technologies generated in the work, or the application of information presented in the manuscript, pose a threat to:

No	Yes	
☐	☐	Public health
☐	☐	National security
☐	☐	Crops and/or livestock
☐	☐	Ecosystems
☐	☐	Any other significant area

Experiments of concern

Does the work involve any of these experiments of concern:

No	Yes	
☐	☐	Demonstrate how to render a vaccine ineffective
☐	☐	Confer resistance to therapeutically useful antibiotics or antiviral agents
☐	☐	Enhance the virulence of a pathogen or render a nonpathogen virulent
☐	☐	Increase transmissibility of a pathogen
☐	☐	Alter the host range of a pathogen
☐	☐	Enable evasion of diagnostic/detection modalities
☐	☐	Enable the weaponization of a biological agent or toxin
☐	☐	Any other potentially harmful combination of experiments and agents

Plants

Seed stocks	Not applicable (see above in Materials & experimental systems section).
Novel plant genotypes	Not applicable (see above in Materials & experimental systems section).
Authentication	Not applicable (see above in Materials & experimental systems section).

ChIP-seq

Data deposition

☐ Confirm that both raw and final processed data have been deposited in a public database such as GEO.

☐ Confirm that you have deposited or provided access to graph files (e.g. BED files) for the called peaks.

Data access links<br>*May remain private before publication.*	*For "Initial submission" or "Revised version" documents, provide reviewer access links. For your "Final submission" document, provide a link to the deposited data.*
Files in database submission	*Provide a list of all files available in the database submission.*
Genome browser session<br>(e.g. UCSC)	*Provide a link to an anonymized genome browser session for "Initial submission" and "Revised version" documents only, to enable peer review. Write "no longer applicable" for "Final submission" documents.*

Methodology

Replicates	*Describe the experimental replicates, specifying number, type and replicate agreement.*
Sequencing depth	*Describe the sequencing depth for each experiment, providing the total number of reads, uniquely mapped reads, length of reads and whether they were paired- or single-end.*
Antibodies	*Describe the antibodies used for the ChIP-seq experiments; as applicable, provide supplier name, catalog number, clone name, and lot number.*

Peak calling parameters | Specify the command line program and parameters used for read mapping and peak calling, including the ChIP, control and index files used.

Data quality | Describe the methods used to ensure data quality in full detail, including how many peaks are at FDR 5% and above 5-fold enrichment.

Software | Describe the software used to collect and analyze the ChIP-seq data. For custom code that has been deposited into a community repository, provide accession details.

Flow Cytometry

Plots

Confirm that:

- [x] The axis labels state the marker and fluorochrome used (e.g. CD4-FITC).
- [x] The axis scales are clearly visible. Include numbers along axes only for bottom left plot of group (a 'group' is an analysis of identical markers).
- [x] All plots are contour plots with outliers or pseudocolor plots.
- [x] A numerical value for number of cells or percentage (with statistics) is provided.

Methodology

Sample preparation

For intracellular flow cytometry:
Murine AML cell lines were harvested, then washed with PBS and stained with Zombie Aqua Fixable Viability Dye (1:1000, BioLegend, San Diego, USA) for 10 minutes. After PBS wash, cells were fixed with 2% phosphate-buffered formaldehyde solution (Roti-Histofix 4,5%, Carl Roth, Karlsruhe, Germany) for 15 min. After washing with PBS, cells were permeabilized with 0.2% TritonX-100 (PanReac AppliChem, Darmstadt, Germany) in PBS supplemented with 10% FBS for 15 min, followed by incubation in 0.1% TritonX-100 in PBS supplemented with 10% FBS for 30 min. Next, cells were incubated with anti-mouse CD16/CD32 antibody (1:200, Mouse BD Fc Block, clone 2.4G2, BD Biosiences, Heidelberg, Germany) for 10 minutes, followed by the direct addition of the 2x primary antibody staining solution (1:400 final dil.) in 0.1% TritonX-100 buffer (V5-Tag, clone D3H8Q, Cell signaling, Danver, USA) and incubation for 45 min. After a wash with 0.1% TritonX-100 buffer, cells were incubated in secondary antibody staining solution (Anti-rabbit IgG AF647, #A-21246, Thermo Fisher Scientific, Waltham, MA, USA) for 45 min. All incubation steps were performed at room temperature with light protection. Stained cells were washed twice with 0.1% TritonX-100 buffer before analysis.

For flow cytometry - cell surface markers:
For characterization of ex-vivo-isolated leukemia blasts, cells from bone marrow and spleen were washed with PBS and resuspended in PBS with 0.5% FCS, followed by staining for 30 min with dilutions (1:200) of the following antibodies (all from Biolegend, San Diego, CA, USA): anti-mouse CD11b/Mac-1 PerCPCy5.5 (clone M1/70), anti-mouse Gr-1/Ly-6C BV421 (clone RB6-8C5) and anti-mouse CD117/c-Kit APC (clone 2B8). Myeloid differentiation was monitored by measurement of surface marker expression as described above using the following antibodies (all from Biolegend, San Diego, CA, USA): anti-mouse CD11b/Mac-1 PerCPCy5.5 (clone M1/70), anti-mouse Gr-1/Ly-6C PeCy7 (clone RB6-8C5) and anti-mouse CD117/c-Kit APC (clone 2B8).

For flow cytometry - apoptosis:
Apoptosis analysis was performed using the Annexin V AF647 Conjugate (#A23204, Invitrogen, eBioscience, Thermo Fisher Scientific) according to the manufacturer's instructions.

For flow cytometry - cell cycle analysis:
For cell cycle analysis, cells were fixed in 70% Ethanol for 30 min before staining with propidium iodide solution for 30 min.

For competitive cell proliferation assays:
To assess the effect of shRNA-mediated gene knockdown on cell proliferation, rtTA3-expressing murine AML cells were transduced with doxycycline-inducible lentiviral shRNA/IRFP670 expression vectors and IRFP670-positive cells were monitored at regular intervals by flow cytometry.

Instrument

For intracellular staining: Stained samples were measured using a FACS Canto II flow cytometer (BD Biosciences), and analyzed with the FlowJo software (FlowJo, LLC).

For flow cytometry: Samples were either measured using a FACS Canto II flow cytometer (BD Biosciences), and analyzed with the FlowJo software (FlowJo, LLC), or measured using an IntelliCyt IQueScreener Plus (BioScience, Sartorius Group, Germany).

For competitive proliferation assays: Cells were monitored at regular intervals using an IntelliCyt IQueScreener Plus (BioScience, Sartorius Group, Germany)

Software | FACSDIVA, FlowJo, ForeCyt

Cell population abundance | No cell sorting was applied, therefore not applicable.

Gating strategy

For all flow cytometry experiments the following gating steps were performed first:
1.) FSC-area vs SSC-area was used to discriminate cells from debris.
2.) FSC-area vs width was used to discriminate singlets.

followed by:

For intracellular staining:
3.) ZombieAqua negative cells were used to discriminate live from dead cells
4.) V5-AF647+ cells were used to monitor NUP98::KDM5A fusion protein levels

For flow cytometry - cell surface markers:
3.) Gates defining positive and negative populations for specific cell surface markers were set according to unstained controls.

For flow cytometry - apoptosis:
3.) AnnexinV-AF647+ cells were used to identify apoptotic cells and the gate was set according to unstained controls.

For flow cytometry - cell cycle analysis:
3.) G1, S and G2/M cell cycle phases were identified in histograms of DNA content according to propidium iodide intensity

For competitive proliferation assays:
3.) iRFP670+ cells were used to identify shRNA-expressing cells.

For flow cytometry - analysis of ex-vivo isolated AML blasts:
3.) CD45.2-PE-Cy7+ cells were used to identify transplanted AML blasts and the gate was set according to unstained controls.
4.) iRFP670+ cells were used to identify shRNA-expressing cells and the gate was set according to iRFP670- controls.
5.) c-Kit-BV421+ cells were used to identify surface marker expression and the gate was set according to unstained controls.

☒ Tick this box to confirm that a figure exemplifying the gating strategy is provided in the Supplementary Information.

Magnetic resonance imaging

Experimental design

Design type: *Indicate task or resting state; event-related or block design.*

Design specifications: *Specify the number of blocks, trials or experimental units per session and/or subject, and specify the length of each trial or block (if trials are blocked) and interval between trials.*

Behavioral performance measures: *State number and/or type of variables recorded (e.g. correct button press, response time) and what statistics were used to establish that the subjects were performing the task as expected (e.g. mean, range, and/or standard deviation across subjects).*

Acquisition

Imaging type(s): *Specify: functional, structural, diffusion, perfusion.*

Field strength: *Specify in Tesla*

Sequence & imaging parameters: *Specify the pulse sequence type (gradient echo, spin echo, etc.), imaging type (EPI, spiral, etc.), field of view, matrix size, slice thickness, orientation and TE/TR/flip angle.*

Area of acquisition: *State whether a whole brain scan was used OR define the area of acquisition, describing how the region was determined.*

Diffusion MRI ☐ Used ☐ Not used

Preprocessing

Preprocessing software: *Provide detail on software version and revision number and on specific parameters (model/functions, brain extraction, segmentation, smoothing kernel size, etc.).*

Normalization: *If data were normalized/standardized, describe the approach(es): specify linear or non-linear and define image types used for transformation OR indicate that data were not normalized and explain rationale for lack of normalization.*

Normalization template: *Describe the template used for normalization/transformation, specifying subject space or group standardized space (e.g. original Talairach, MNI305, ICBM152) OR indicate that the data were not normalized.*

Noise and artifact removal: *Describe your procedure(s) for artifact and structured noise removal, specifying motion parameters, tissue signals and physiological signals (heart rate, respiration).*

Volume censoring | *Define your software and/or method and criteria for volume censoring, and state the extent of such censoring.*

Statistical modeling & inference

Model type and settings | *Specify type (mass univariate, multivariate, RSA, predictive, etc.) and describe essential details of the model at the first and second levels (e.g. fixed, random or mixed effects; drift or auto-correlation).*

Effect(s) tested | *Define precise effect in terms of the task or stimulus conditions instead of psychological concepts and indicate whether ANOVA or factorial designs were used.*

Specify type of analysis: ☐ Whole brain ☐ ROI-based ☐ Both

Statistic type for inference | *Specify voxel-wise or cluster-wise and report all relevant parameters for cluster-wise methods.*

(See Eklund et al. 2016)

Correction | *Describe the type of correction and how it is obtained for multiple comparisons (e.g. FWE, FDR, permutation or Monte Carlo).*

Models & analysis

n/a	Involved in the study
☐	☐ Functional and/or effective connectivity
☐	☐ Graph analysis
☐	☐ Multivariate modeling or predictive analysis

Functional and/or effective connectivity | *Report the measures of dependence used and the model details (e.g. Pearson correlation, partial correlation, mutual information).*

Graph analysis | *Report the dependent variable and connectivity measure, specifying weighted graph or binarized graph, subject- or group-level, and the global and/or node summaries used (e.g. clustering coefficient, efficiency, etc.).*

Multivariate modeling and predictive analysis | *Specify independent variables, features extraction and dimension reduction, model, training and evaluation metrics.*
